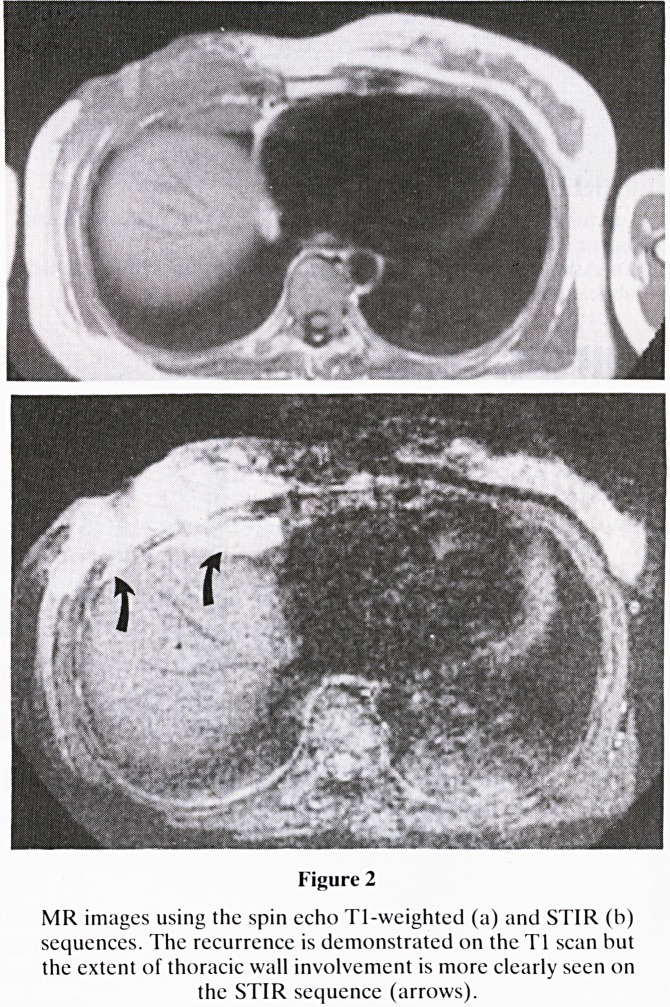# Magnetic Resonance Imaging and Carcinoma of the Breast

**Published:** 1989-05

**Authors:** P G Cook, P Goodard

**Affiliations:** Consultant Radiologists, Bristol Royal Infirmary; Consultant Radiologists, Bristol Royal Infirmary


### Magnetic Resonance Imaging and Carcinoma of the Breast

**P G Cook and P Goddard**
Consultant Radiologists, Bristol Royal Infirmary

Mammography remains the main investigation in the detection of breast carcinoma, both for screening and where clinical suspicion of the tumour exists. Once a tumour of the breast has been diagnosed using mammography, staging of the tumour occurs. This may involve surgical removal of the tumour and axillary lymph nodes as the first procedure, but more frequently further radiological techniques are used to determine the extent and site of any spread. The most often used additional investigations are the plain chest film, followed by the isotope bone scan. These methods detect much of the metastatic disease as the chest and bones are major sites of metastases. Problems do arise however, in detecting local spread involving the chest wall and in those areas where metastases can occur alongside other disorders which may also appear abnormal on the investigations used currently. This is particularly true in the spine where a bone scan may demonstrate 'hot spots' associated with degenerative disase, infection, osteoporotic crush fractures and previous surgery. These 'hot spots' may also remain as such for many months.

Computed tomography has helped in the detection of both local spread and distant spread to the chest, particularly mediastinal nodes, and to the spine. This technique also has its limitations. It has been shown, in the absence of bone destruction, to be insensitive in the detection of chest wall involvement by tumours. Further, the detection of metastatic disease of the spine may require many axial 'slices' to examine the relevant areas of suspicion. Mediastinal node enlargement may also prove to be difficult to detect in some cases, without the use of a large bolus of contrast medium to delineate vessels.

Magnetic Resonance Imaging has been made available more recently and is demonstrating its use in many areas. It is already an established tool in the investigation of the central nervous system. It has shown its use in the evaluation of bone tumours and other skeletal abnormalities more recently. Currently it is showing great promise in the investigation of many primary tumours including breast carcinoma and sarcoma.

To demonstrate the use of MRI in the investigation of breast tumours two cases are briefly described.

**Figure 1**

T1 weighted MR scan demonstrating reduced signal intensity in the vertebral body affected by metastatic disease and a small area of similar signal intensity in L3 (arrow).

**Figure 2**

MR images using the spin echo T1-weighted (a) and STIR (b) sequences. The recurrence is demonstrated on the T1 scan but the extent of thoracic wall involvement is more clearly seen on the STIR sequence (arrows).

### CASE 1

A 50 year old woman with known carcinoma of the breast who presented with backache. An isotope bone scan had demonstrated an isolated hot spot in the upper lumbar spine. An MR scan was performed and demonstrated unequivocal evidence of metastasis of L2 and a small unsuspected metastasis in L3 (figure 1).

### CASE 2

A 34 year old woman with a sarcoma of the right breast who had undergone a mastectomy and resection of a recurrence. The extent of the recurrence was not known. An MR scan was performed and demonstrated unequivocal invasion through the thoracic wall, extending to the right cardiac border (figure 2).

## Figures and Tables

**Figure 1 f1:**
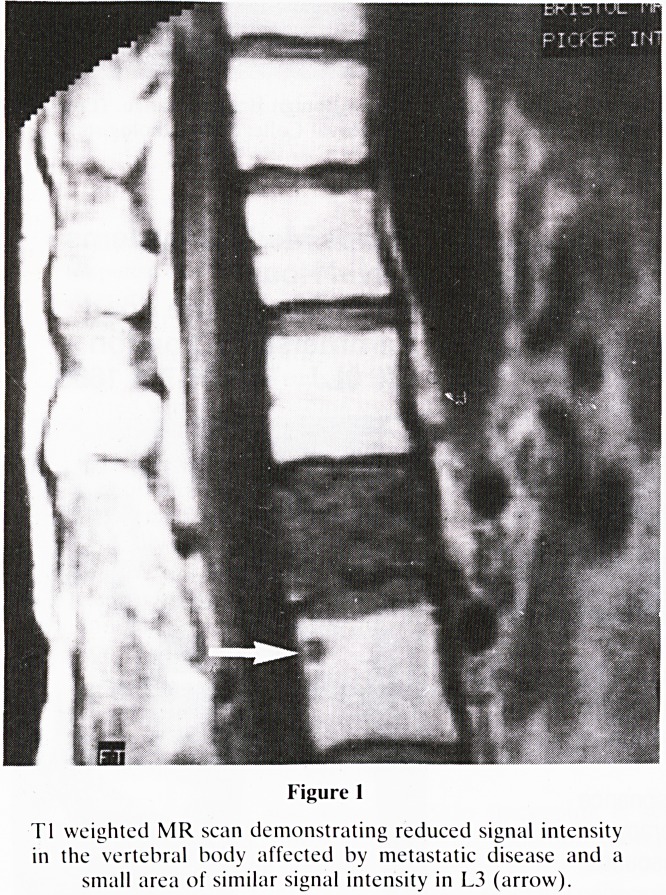


**Figure 2 f2:**